# Combine Effect of ZnO NPs and Bacteria on Protein and Gene’s Expression Profile of Rice (*Oryza sativa* L.) Plant

**DOI:** 10.3390/toxics10060305

**Published:** 2022-06-03

**Authors:** Nazneen Akhtar, Sehresh Khan, Muhammad Jamil, Shafiq Ur Rehman, Zia Ur Rehman, Eui Shik Rha

**Affiliations:** 1 Department of Biotechnology and Genetic Engineering, Kohat University of Science & Technology (KUST), Kohat 26000, Pakistan; nazneen_kht92@yahoo.com (N.A.); sehreshkhan91@yahoo.com (S.K.); ziamarwat77@gmail.com (Z.U.R.); 2 Department of Biology, University of Haripur, Haripur 22620, Pakistan; drshafiq@yahoo.com; 3 Department of Well-Being Resources, Sunchon National University, Suncheon 540-742, Korea

**Keywords:** heavy metals, nanoparticles, transcription factors, gel electrophoresis, zinc oxide nanoparticles, bacteria

## Abstract

Heavy metal (HM) emissions have increased due to the impact of rising urbanization and anthropogenic activity, affecting different parts of the environment. The goal of this study is to investigate the combined effect of ZnO NPs and bacteria treatment on protein and gene expression profiles of rice plants that are grown in HMs-polluted water. Seeds were primed with *Bacillus* spp. (*Bacillus cereus* and *Lysinibacillus macroides*) before being cultured in Hoagland media containing ZnO NPs (5 and 10 mg/L) and HMs-contaminated water from the Hayatabad industrial estate (HIE), Peshawar, Pakistan. The results revealed that the maximum nitrogen and protein content was observed in the root, shoot, and leaf of the plant grown by combining bacteria-ZnO NPs treatment under HMs stress as compared with plant grown without or with individual treatments of ZnO NPs and bacteria. Furthermore, protein expression analysis by sodium dodecyl sulfate polyacrylamide gel electrophoresis (SDS PAGE) revealed that plants that were grown in HMs-polluted water were found to be affected in contaminated water, however the combined effect of bacteria-ZnO NPs reported the more dense protein profile as compared with their individual treatments. Subsequently, plants that were grown in HMs-polluted water have the highest expression levels of stress-induced genes such as myeloblastosis (*Myb*), zinc-finger protein (*Zat*-12), and ascorbate peroxidase (*Apx*) while the combined effect revealed minimum expression as compared with individual treatments. It is concluded that the combined effect of ZnO NPs and bacteria lowered the stress-induced gene expression while it increased the nitrogen-protein content and protein expression in plant grown under HMs stress.

## 1. Introduction

In recent years, there has been a huge interest in the proteomic and molecular mechanisms of plant interaction with environment. Plants are continually exposed to harmful environmental conditions throughout their life cycle [1]. The presence of toxic compounds such as heavy metals (HMs), significantly damage the plant by altering the plant’s physiology and metabolic processes [2]. A recent study by Akhtar [3] revealed that the water samples that were obtained from Hayatabad industrial estate (HIE), Peshawar and Gadoon industrial estate (GIE), Sawabi, Pakistan, contain large amounts of HMs that are further used to irrigate local vegetable crops. Since plants are sessile, they are often subjected to a wide range of biotic or abiotic stresses, including HM stress [4].

Rice (*Oryza sativa* L.) super basmati cv. (pedigree from Basmati-320) is the main staple food that is used for more than 2.7 billion people all around the world. Basmati rice is characterized by the extra-long slender grain, pleasant and distinct aroma, and soft and fluffy texture of the cooked rice [5]. Rice plants are submerged for a long period of time in water so there is a high chance of HMs accumulation in the plant. Nitrogen is one of the most abundant macronutrients that is utilized by rice plants, however HMs dramatically decrease nitrogen absorption and assimilation, changing nitrogen metabolism. Proteins are functionally diverse macromolecules that act as the main building blocks of living cells [6]. Furthermore, the conformation of a protein is heavily influenced by reactive molecules, heavy metal (HM) ions, and other stresses [7]. HMs-induced changes weaken the stable interactions of the tertiary structure and result in the loss of the protein’s functions, cell signaling proteins, transportation proteins, and affect the regulation and catalysis processing plant [8], disrupting the protein folding by causing an aggregation of proteins, changing the cell viability and damaging the endoplasmic reticulum, and reducing the nitrogen uptake, assimilation, and metabolism [9].

HMs significantly change the expression of these stress-induced genes by different cell signaling pathways such as a receptor-coupled phosphorylated pathway and mitogen-activated protein kinase and damage the DNA and chromosome aberration. Stress-induced genes such as ascorbate peroxidase (APX), myloblastosis (MYB), and zinc-finger protein (ZAT-12) have uncontrolled expression in plants that are under abiotic stress [10]. These TFs are up-regulated in plant during stress and control the different metabolic, physiological and biochemical process in plants by the activation and repression of transcription processes. The *Myb* transcription factor gene family is expressed during the growth, differentiation processes, and under stressful conditions in rice plants [11]. Zinc-finger protein (Zat-12) is the large network of functional and regulatory genes that are expressed during biotic and abiotic stress in plants [12]. Furthermore, these toxic metals also produce oxidative stress, which results in the formation of reactive oxygen species (ROS), that cause extensive cell damage and inhibit photosynthetic processes. Plants adopt different enzymatic and non-enzymatic mechanisms to neutralize the oxidative stress and toxicity of HMs [13]. Ascorbate peroxidase (APX) is one of the anti-oxidative enzymes that plays an important role in plants during oxidative stress and converts H_2_O_2_ into H_2_O and O_2_ by Haem peroxidases enzymes [14]. There are eight different types of APX genes have been reported in rice plants (*Oryza sativa* L.) that show maximum expression during HMs stress. These types include two cytosolic genes (Os APx1 and Os APx2), two putative peroxisomal genes (Os APx3 and Os APx4), and four chloroplast isoform genes (OsAPx5, OsAPx6, OsAPx7, and OsApx8) [11].

Bacteria-mediated seed priming is aimed to control seed hydration by lowering the external water potential or shortening the hydration period during stress. Several bacteria species, including *Bacillus* spp. were shown to solubilize zinc ions throughout growth by forming complexes with protons [11] and meet nutritional needs by using zinc ions as a cofactor in their metabolic process [12]. However, seed bio-priming is insufficient to improve seed growth in a toxic environment, and the solubilized type of HMs in water cannot be easily extracted by bacteria. Bio-nanotechnology has created a bond between bacteria and nanoparticles, so at a low cost and with high efficiency, nanoparticles at lower doses improved bacterial resistance to HMs. Zinc oxide nanoparticles are used as non-fertilizers that act as important adsorbents for remediation, due to the fact that they carry various functional hydroxyl groups and protons on their surfaces [13]. Furthermore, zinc defends plant cell organelles from oxidative stress and functions as a participant in the plant’s defense system [14,15]. Bio-primed seeds (seeds with bacterial inoculum) growing in ZnO NPs solution are a simple, and easily adaptable strategy to mitigate stress and improve the germination of seeds. In synergistic treatment, bacterial priming gives a protective coat around the seeds to prevent the entry by (1) producing auxin (IAA) hormones and activating cell division, (2) stabilizing the bio-membranes integrity, (3) phospholipid formation, (4) increasing protein synthesis, (5) remediating oxidative stress, (6) transferring the nutrients from aged cells to newborn cells, and (7) lowering the uptake [11]. Furthermore, Since ZnO NPs are much more stable and also have a longer life, they change into Zn^+2^ ions at lower doses, and bacterial cells inactivate these ions in the media by cells that secrete specific metabolites and organic acids [12]. These zinc ions have helped plant growth by strengthening the membrane, macromolecules, different steroid receptors, and carbohydrate metabolism, further eliminating the harmful effects of HMs in water [13]. 

As such, in the past, the individual effect of nanoparticles and bacteria under heavy metals have been studied extensively [16] but no data are available about the synergistic impact of ZnO NPs and bacteria-primed seeds on protein and gene expression in rice seedlings. Therefore, the present study looked at the impact of combining bacteria and ZnO NPs on plant growth in metal-stressed conditions. Rice (*Oryza sativa* L.) seeds were prepared with *Bacillus* spp. and grown in hydroponic culture trays with lower doses of ZnO NPs and HMs-polluted water. As a result, this study confirms the importance of the combined effect of *Bacillus* spp. and ZnO NPs in the variation of protein and gene profiles of rice plants under HM stress.

## 2. Materials and Methods

### 2.1. Water Sampling

Wastewater samples were collected from Hayatabad industrial estate (HIE) that is present near the suburban town of Hayatabad Industrial Estate (HIE) Peshawar, Pakistan. The samples were obtained in clean and dry bottles and stored at 4 °C to analyze the physicochemical properties. The heavy metal content in the Hayatabad industrial estate (HIE) water was determined by following the methodology as described previously by Radulescu et al. [17]. The HMs content was analyzed by adding 2 mL of concentrated nitric acid (HNO_3_) and 5 mL of hydrochloric acid (HCl) in the water samples and boiling at 95 °C in a water bath. The water was heated until the volume reduced to 15–20 mL. The HMs content was then analyzed by atomic absorption spectroscopy (Perkin Elmer Waltham, Massachusetts, United States, Analyst 4000). The physicochemical properties and heavy metal concentration of the HIE water were analyzed to determine the pollution load and then it was compared with the standard value of National Environmental Quality Standards (NEQS, 2000). NEQS is the uniform standard for industrial and municipal effluents use for irrigation purposes.

### 2.2. Characteristics of ZnO NPs

ZnO nanoparticles were collected from Pir Mehr Ali Shah (PMAS) arid agriculture university, Pakistan. It was synthesized by co-precipitation method by using the protocol of Hussain et al. [18] with some modification. ZnO nanoparticles were prepared by mixing 0.05 M Zinc acetate Zn(NO_3_)_2.6_H_2_O (25 mL) solution with 4 mL of plant extract. Solution was heated and continuously stirred. The solution was centrifuged at 12,000 rpm for 15 min. Supernatant was discarded, and the isolated pellet was again suspended in deionized water. The solution was again centrifuged for 5 min and repeated the process three times to remove impurities. Synthesized ZnO NPs were white in color. The stock solution of nanoparticles was stabilized at pH 10 to maintain their activity.

ZnO NPs were characterized by field emission scanning electron microscopy (FESEM), transmission electron microscopy (TEM), Fourier transform infrared spectroscopy (FTIR), and X-ray diffraction (XRD). The X-ray diffraction studies of ZnO NPs were carried out using Rigaku 600 Miniflex X-ray diffraction instrument (XRD) with Cukα radiation (λ = 1.5412 Å) in the scanning range of 100–800. To confirm the absorbance of ZnO NPs and to observe the changes in the absorbance that are caused due to variations in reaction conditions, UV-visible (UV-vis) spectra were carried in the wavelength range of 200–600 nm using Agilent Technologies Cary 60 UV-vis. The ZnO NPs were range from 30–50 nm in size [19].

### 2.3. Rice Plant

Rice (*Oryza sativa* L. cv Super Basmati) was obtained from the National Agriculture Research Center (NARC), Islamabad, Pakistan. The seeds were surface sterilized with a 1% solution of sodium hypochlorite (NaOCl) and rinsed thoroughly with deionized water.

### 2.4. Bio-Priming of Seeds

A total of two heavy metal-resistant bacterial strains *Bacillus cereus* (PMBL-3) and *Lysinibacillus macroides* (PMBL-7) was isolated from wastewater of the Gadoon industrial estate, Khattak [20] and were collected from the Plant and Microbial Biotechnology Lab, Kohat University of Science & Technology (KUST), Kohat, Pakistan. Cell suspension (107 to 109 CFU/mL) of the bacteria strains were grown at 37 °C for 24 h. The seeds were primed with *B. cereus* and *L. macroides* strains by adding 2% sucrose for 10 h in dark conditions [21].

### 2.5. Hydroponic Culture Experiment

Firstly, the seeds were primed with the bacterial strains (*B. cereus* and *L. macroides*) and were grown for 10 days in distilled water (DW) [22]. After 10 days, the young seedlings were grown in 3 L hydroponic system containing one-fourth strength of Hoagland solution. For example, it is clear from the, in which the rice seeds that were primed with the bacterial strains (*B. cereus* and *L. macroides*) that were grown in the hydroponic culture experiment contained ZnO NPs solutions along with HMs-contaminated water. The trays were covered with packing material to keep the root area dark. The experiment included 9 treatments of distilled water grown plants as a control. After 21 days, the plants were moved from the Hoagland tray to a separate tray which was filled with 5 and 10 mg/L of ZnO NP solutions along with HMs-polluted wastewater (w.w) of the Hayatabad industrial estate (HIE). In this analysis, the HM concentrations were chosen based on the average observed values of Pb, Cd, Cr, and Cu in agricultural land [23]. Several previous studies have reported that ZnO NPs at 5 and 10 mg/L had no phytotoxic impact on plant seedlings [24]. The Hoagland solution was prevented during the exposure phase, due to a high ion concentration of nutrients that caused an accumulation of ZnO NPs. The hydroponic method has been used to control the solubility of ZnO NPs, NPs, and HMs [25]. A total of three replicates of each treatment as well as a blank control were grown. During the exposure period, plants were rotated and moved at random to ensure equal light exposure. At the end of the HMs / ZnO NPs treatment, the pH of the growing medium was measured with a pH meter.

### 2.6. Growth Analysis and Partitioning of Biomass

The root biomass was isolated from the water tank and washed 5 times with 5 mM of 50 mL CaCl_2_ solution to remove the zinc and HMs components that had been deposited on the plant root for the exposure experiment (buffer solution). The growth media was collected to determine the HMs content while the plants were harvested and divided into fresh and dry material. Half of the fresh plant material was stored at −70 °C for proteomic and molecular analysis while others were dried at 80 °C in an oven for 5 days to confirm that the tissues had been fully dehydrated [26]. The experiment contained three replicates of each plant treatment.

### 2.7. Total Nitrogen-Protein

The total nitrogen and protein content of plant tissues was determined using the Kjeldahl apparatus (Perkin Elmer, Analyst 4000, Waltsman, MA, USA) by following the methodology as specified previously by Saez-plaza [27]. The dried plant samples (100 mg) were digested with digestion mixture (10 mL) in a digestion assembly. Subsequently, 2 mL of concentrated sulfuric acid (H_2_SO_4_) was added in mixture and incubated until a clear solution was formed. Subsequently, the digested mixture was then transferred in the distillation assembly and 4 mL of 2% sodium hydroxide (*w*/*v*)) and 50 mL of 1% boric acid (Thermo Fisher Scientific, Bedford, MA, USA) was added. After that, 20 mL of distilled water (DW) was added to the solution. One drop at a time of methyl red was added to the sample, and the sample was titrated with H_2_SO_4_ before the normality level was reached. The total protein-nitrogen contents were determined by the given formula

$$\begin{array}{c} \mathrm{Total}\;\mathrm{nitrogen}\;\left(g/g\right)\\ =\frac{\mathrm{Volume}\;\mathrm{of}\;\mathrm{sample}\;-\;\mathrm{Volume}\;\mathrm{of}\;\mathrm{blank}\;\times \;0.1N}{\mathrm{Dry}\;\mathrm{weight}\;\mathrm{of}\;\mathrm{sample}}\\ \times 100 \end{array}$$

Total protein (g/g) = Total nitrogen % × 6.25

(Protein factor = 6.25, Nitrogen factor = 1.4007)

### 2.8. Total Structural Protein

#### 2.8.1. Protein Extraction from a Plant Sample

The protein expression in SDS-PAGE was determined by the protocol of Laemmli [28]. Firstly, 400 µL of protein extraction sample was mixed with 15 mg of fresh plant content and pH 8 was maintain. The protein extraction buffer consisted of 1 ml of 2.5% sodium-dodecyl sulphate (SDS), 100 µL of 0.5 M Tris-HCl, 100 µL of 5% 2-marcaptoethanol, 100 µL of 10% glycerol, and a small concentration of bromophenol blue (BPB) (Sigma-Aldrich, St. Louis, CA, USA). Firstly, the mixture was vortexed for 7 min and kept at 40 °C for 24 h. After that, the samples were centrifuged at 13,000 rpm for 10 min and the supernatant was stored at 4 °C to remove the protein.

#### 2.8.2. Bradford Assay

The protein samples were tested using the Bradford assay [29].

#### 2.8.3. SDS-PAGE, (Sodium, Dodecyl, Sulfate, Polyacrylamide Gel Electrophoresis) Analysis

The expression of the protein samples were studied using the SDS-PAGE system (Sigma-Aldrich, St. Louis, CA, USA). There were two separate gels (4% stacking gel and a 12% resolving gel) that were used to isolate the proteins (Table 1). In each well, an equal amount (12 µL) of each sample was filled while 5 µL of protein ladder (fermentase) (Thermo Fisher Scientific, Bradford, MA, USA) with molecular weights ranging around 10 to 200 kDa were loaded in one of the wells. The gel was run at 120 volts for 120 min in the electrophoresis tank. The bands in each well were observed by soaking the gel for 40 min in a 50 mL of staining solution. The gel was placed in a destining solution for 5 h, and clear bands were identified using the gel documentation process (Thermo Fisher Scientific gel documentation system).

### 2.9. Molecular Studies

#### 2.9.1. RNA Extraction

An RNA extraction method was used to separate RNA from the fresh plant leaves as previously described by Deepa [30]. Firstly, the plant material was mixed with 1 mL of trizole (Sigma-Aldrich, St. Louis, CA, USA) and vortexed for 15 min. The materials were completely mixed until being heated for 10 min (RT). The samples were centrifuged at 13,000 rpm for 10 min to remove plant debris. After that, 2 mL of supernatant was mixed with 200 µL of chloroform (Thermo Fisher Scientific, Bedford, MA, USA) in a separate tube. Subsequently, the supernatant was placed for 15 min at cold temperature. The samples were centrifuged for 15 min at 13,000 rpm in a 4 °C pre-cooled centrifuge for phase separation. The aqueous phase (50–60% trizol) was transferred to separate tubes and added 5 mL of isopropanol in mixture (Thermo Fisher Scientific, Bedford, MA, USA). After mixing, the tube was incubated at 37 °C for 10 min. The cells were filtered again at 13,000 rpm for 10 min. The pellets are immersed in RNase water and the sample was kept at −20 °C.

#### 2.9.2. cDNA Synthesis

Fresh RNA was extracted and oligo (dT) primers (Thermo Fisher Scientific, Bedford, MA, USA) were used to make cDNA (Thermo, scientific, revert aid first starting cDNA synthesis kit). Actin is a housekeeping gene that is always expressed and serves as a regulatory element in plants.

#### 2.9.3. Polymerase Chain Reaction (PCR)

Primers from different gene families, such as ascorbate peroxidase (APX), myeloblastosis (MYB), and zinc finger protein (Zat-12) were used to amplify cDNA in a thermocycler (Applied Bio systems, Foster city, CA, USA). The PCR conditions were as follows: (pre-denaturation at 95 °C for 5 min, 30 cycles, (95 °C for 20 s), (60 °C for 30 s) and (72 °C for 4 s) and the final temperature was (72 °C for 12 min). The samples were then run on a 1.5% agarose gel. e. Using a gel documentation method, the difference in the bands was observed (Thermo Fisher Scientific gel documentation system, MA, USA).

### 2.10. Statistical Analysis

The statistical analysis was performed using the Statistic 9 software (v.10, Informer Technologies, Inc., Los Angeles, CA, USA), and the variance between the different treatments was determined by least significance difference (LSD) multiple comparisons and an ANOVA test. The probability level of (*p* ≤ 0.05) was used to determine statistically significant variance.

## 3. Results

### 3.1. Physicochemical Properties of Wastewater

The physicochemical properties of the Hayatabad industrial estate (HIE) water samples were analyzed to determine the pollution load and then compared with the standard value of National Environmental Quality Standards (NEQS, 2000). Temperature is a significant parameter of water for the survival of aquatic organisms, so the observed temperature was 24.5 °C which is below the permissible limit of NEQS (40 °C). The nature of water was basic and the observed pH was 7.23 which was in the permissible limit pH (6–10) for NEQS. The electrical conductivity of the water was 682 µS/m which was above the limit of 500 µS/m. It was found that the total-suspended-solids (TSS) were 400 mg/L which was higher than the permissible limit of NEQS (150 mg/L). The total-dissolved-solids (TDS) in industrial effluents were 4485 mg/L which was above the permissible limits of NEQS (3500 mg/L) respectively. The biochemical oxygen demand (BOD) in industrial effluent was 250 mg/L which was above the permissible limit of 80 mg/L. the chemical oxygen demand (COD) in the industrial effluents was 400 mg/L, which was above the limits of NEQS (150 mg/L). It was also observed that the HMs in the industrial effluents were more than the limits of the NEQS standards for irrigation purposes (Table 2).

### 3.2. Total, Nitrogen and Protein Content

The results revealed that plants that were grown in HMs-polluted water showed lowered nitrogen (2.21, 1.621, and 1.12 µg/g) and protein (15.32, 9.23, and 6.21 µg/g) content in the leaf, shoots, and roots of plants that were cultivated in filtered distilled water (5.62, 4.231, and 3.11 µg/g), (45.43, 40.43, and 32.22 µg/g), respectively. In the HMs-polluted water, there was maximum nitrogen and protein content in the leaf-shoot-root of growing plants with seeds that were primed with *B. cereus* (25.32, 20.43, and 17.62 µg/g), (32.21, 26.54, and 16.43 µg/g) and *L. macroides* (22.65, 17.22, and 15.51 µg/g) (30.22, 22.21, and 14.21 µg/g) and grown at 5 mg/L ZnO NPs in contrast to plant only growth with *B. cereus* (14.32, 10.52, and 8.23 µg/g) (23.21, 16.43, and 10.23 µg/g) and *L. macroides* (12.13, 8.32, and 6.43 µg/g) (21.2, 14.2, and 9.23 µg/g) in the absence of ZnO NPs and in the presence of 5 mg/L ZnO NPs (12.23, 7.21, and 5.46 µg/g) (22.34, 16.54, and 10.73 µg/g), respectively (Table 3).

### 3.3. Total Structural Protein (SDS-PAGE)

The protein content of each sample was defined by the protocol that was outlined previously by Bradford [29]. The standard curve of bovine serum albumin was used to determine the quantity of protein in the plant samples (BSA). After that, SDS-PAGE was used to assess the protein expression in each treatment.

**Figure 1:**
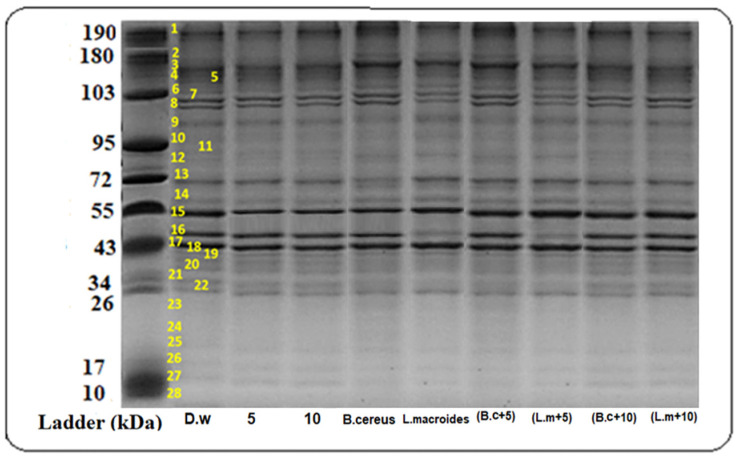
Protein banding profiles were detected on SDS-PAGE with CBB staining of rice plants that were grown by the synergistic treatment of seeds that were primed with bacteria and grown in ZnO NPs alone or in combination with distilled water. (Lane 1: DW (distilled water), Lane 2: Bio-priming with *Bacillus cereus*, Lane 3: Bio-priming with *Lysinibacillus macroides*, Lane 4: 5 mg/L ZnO NPs treatment, Lane 5: 10 mg/L ZnO NPs treatment, Lane 6: *Bacillus cereus* + 5 mg/L ZnO NPs, Lane 7: *Lysinibacillus macroides* + 5 mg/L ZnO NPs, Lane 8: *Bacillus cereus* + 10 mg/L ZnO NPs, Lane 9: *Lysinibacillus macroides* + 10 mg/L ZnO NPs).

**Figure 2:**
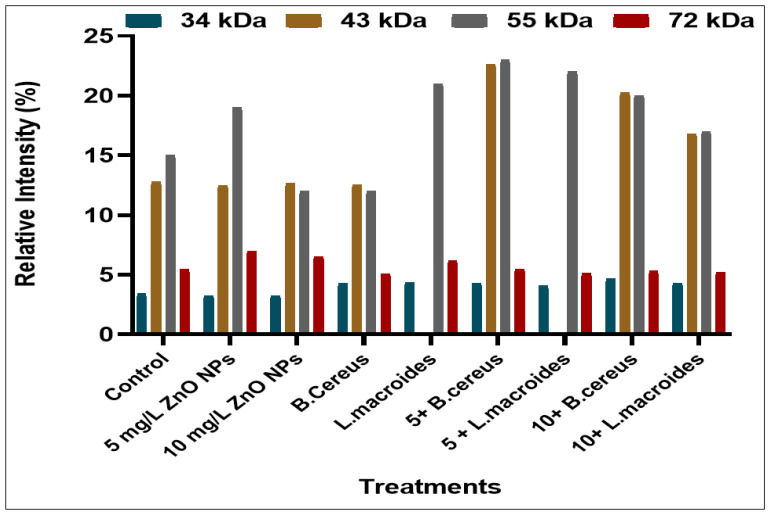
Protein banding intensity of rice plants that were grown by the synergistic treatment of seeds that were primed with bacteria and grown in ZnO NPs alone or in combination with distilled water at marker size (34, 43, 55, and 72 kDa).

Various intensities of the protein bands were observed in plants that were grown in distilled water at different molecular weights (34, 43, 55, 72, 130, 180, and 190 kDa) (Figure 1) while the maximum protein bands at marker size (34, 43, 55, 72 kDa) with banding intensity (4.286, 22.692, 23.00, and 5.490%) (Figure 2) was observed in the combined impact of *B. cereus*-primed seeds grown at 5 mg/L ZnO NPs. It was reported that the protein bands that were present at these marker sizes contained enzymes such as peroxidases, nucleases, RNA polymerases II, and lipo-oxygenase [31,32]. These proteins showed a maximum expression as compared with proteins that was found in plants that developed from *B. cereus-*primed seeds with a banding intensity without ZnO NPs treatments (4.235, 12.541, 12.00, and 5.079%) and grown in (5 mg/L) ZnO NPs with a banding intensity (3.262, 12.466, 19.00, and 6.982%), respectively. Dense bands with higher banding intensity were observed in *L. macroides*-primed seeds that were grown at 5 mg/L ZnO NPs (4.057, 22.121, and 5.21%). According to the literature, there was peroxidases, nucleases, RNA polymerases II, and lipooxygenase protein present with intensity at these marker sizes as opposed to the protein that was present in individually-primed seeds without ZnO NPs treatments (4.235, 21.00, and 6.210%) with 5 mg/L ZnO NPs treatments (3.262, 12.46, 19.01, and 6.983%) respectively.

**Figure 3:**
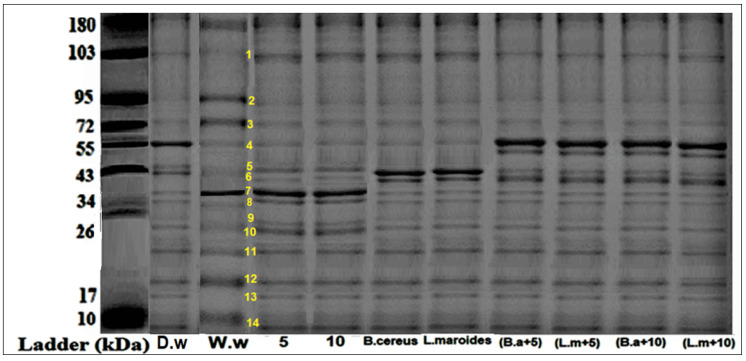
Protein banding profile that was detected on SDS-PAGE with CBB staining of rice plants that were grown by the synergistic treatment of seeds that were primed with bacteria and grown in ZnO NPs alone or in combination with HMs-contaminated water. (Lane 10: Wastewater (w.w), Lane 11: bio-priming with *Bacillus cereus*+ w.w, Lane 12: bio-priming with *Lysinibacillus macroides* + w.w, Lane 13: 5 mg/L ZnO NPs + w.w treatment, Lane 14: 10 mg/L ZnO NPs + w.w treatment, Lane 15: *Bacillus cereus*+ 5 mg/L ZnO NPs + w.w, Lane 16: *Lysinibacillus macroides* +5 mg/L ZnO NPs + w.w, Lane 17: *Bacillus cereus*+ 10 mg/L ZnO NPs + w.w, Lane 18: *Lysinibacillus macroides* +10 mg/L ZnO NPs + w.w).

**Figure 4:**
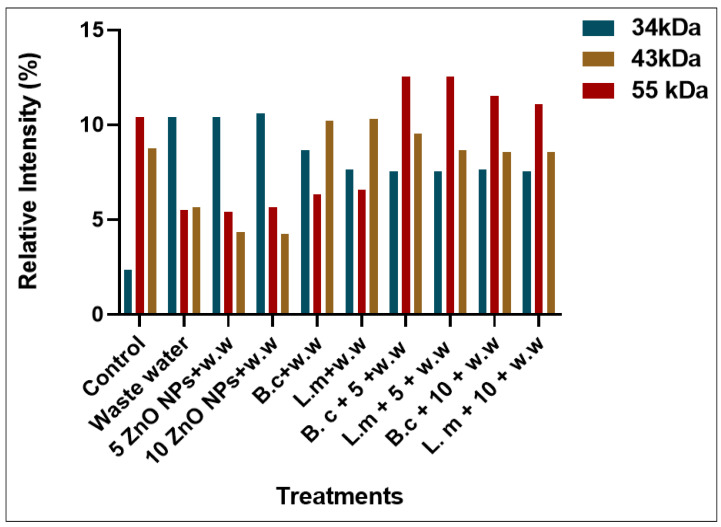
Protein banding intensity of rice plants by the synergistic treatment of seeds that were primed with bacteria and grown in ZnO NPs alone or in combination with heavy metal-contaminated water at marker size 34, 43 and 55 kDa.

Plants that were grown in the HMs-polluted water showed the lowest expression of protein bands at different marker sizes (34, 43, 55, 72, 130, 180, and 190 kDa) (Figure 3 and Figure 4). Those plants that were grown primed with *B. cereus* with 5 mg/L of ZnO NPs showed the maximum expression of bands with banding intensity 7.54, 9.540, and 12.55% at marker size 34, 43, 55 kDa, respectively. There was evidence in the literature that there were peroxidases, nucleases, cellulose synthesis proteins that were present at these marker sizes. These proteins are much effective comparison to the *B. cereus*-primed seeds without ZnO NPs (8.65, 10.23, and 6.34%) and one grew in 5 mg/L ZnO NPs (10.40, 4.340, and 5.430%), respectively. Furthermore, maximum bands were observed in plants that were grown by the seeds that were primed with *L. macroides* and grown at 5 mg/L of ZnO NPs (7.532, 8.65, and 12.54%) revealed peroxidases, nucleases, cellulose synthesis proteins as compared with plants that were grown by the primed seeds without ZnO NPs treatments (7.32, 10.32, and 6.56%) and grown in 5 mg/L ZnO NPs (10.40, 4.340, and 5.430%).

### 3.4. Molecular Analysis

The synergistic treatment of bacteria and ZnO NPs in HMs-polluted water showed gene family expression profiles, such as myeloblastosis (*Myb*), zinc finger protein (*Zat*-12), and ascorbate peroxidase (*Apx*) in growing plants.

#### 3.4.1. Expression of Myeloblastosis (Myb) Gene

**Figure 5:**
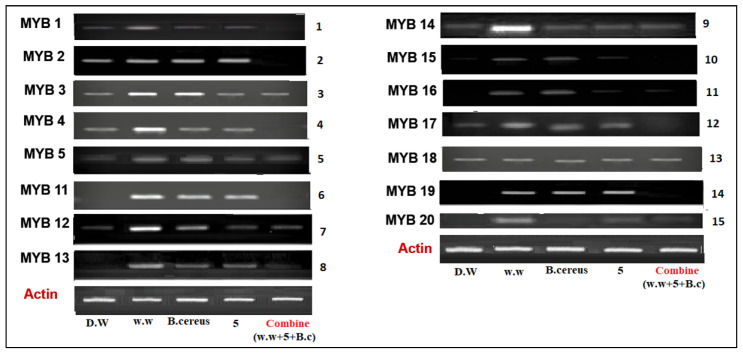
The expression profile of Myeloblastosis (MYB) gene family of rice plants that were grown by the synergistic treatment of bacteria-primed seeds that were grown in 5 mg/L ZnO NPs and HMs-contaminated water. Treatments are DW (distilled water), w.w (wastewater), bio-priming (*Bacillus cereus* + w.w), ZnO NPs treatment (5 mg/L + w.w), and combined treatments (*Bacillus cereus*+ 5 mg/L ZnO NPs + w.w).

**Figure 6:**
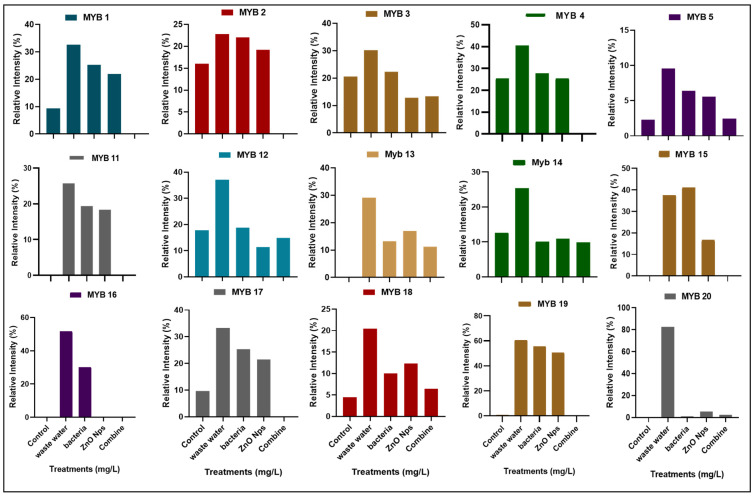
Relative intensity (%) of the myeloblastosis (MYB) gene family of rice plants that were grown by the synergistic treatment of bacteria-primed seeds that were grown in 5 mg/L ZnO NPs and HMs-contaminated water. The treatments were DW (distilled water), w.w (wastewater), bio-priming (*Bacillus cereus* + w.w), ZnO NPs treatment (5 mg/L + w.w), and combined treatments (*Bacillus cereus* + 5 mg/L ZnO NPs + w.w).

The *Myb* gene has an important role in plant growth and development. The *Myb* gene family contains 20 genes in rice plants that function as transcription factors. In the current research, we investigated the expression of 15 *Myb* genes in plants in different conditions, such as the expression of genes in plants that were grown in control conditions (distilled water), HMs polluted water, individual treatments of *B. cereus*-primed seeds and 5 mg/L ZnO NPs, and the synergistic treatment of *B. cereus*-primed seeds and 5 mg/L ZnO NPs (Figure 5). Plants that were grown in HMs-polluted water in gene families *Myb*3, *Myb*4, *Myb*11, *Myb*12, *Myb*13, *Myb*14, and *Myb*19 showed the maximum expression with the maximum banding intensity (30.24, 40.59, 25.74, 37.118, 29.156, 25.35, and 60.542%, respectively) in comparison with the control-grown (distilled water) plants while the plants showed the lowest amount of expression of *Myb*2, *Myb*3, and *Myb*4 with banding intensity 22.754, 30.241, and 40.594%, respectively. When we related our results to that of the other treatments, we found that seeds that were primed with bacteria and grown in 5 mg/L ZnO NPs had no expression of *Myb*1, *Myb*2, *Myb*4, *Myb*11, *Myb*15, *Myb*17, and *Myb*19 as compared with single-primed seeds that showed minimum expression with banding intensity (25.33, 22.076, 27.748, 19.29, 37.454, 25.35, and 55.54%), respectively (Figure 6).

#### 3.4.2. Expression of Zinc Finger Protein (Zat-12) Gene Family

**Figure 7:**
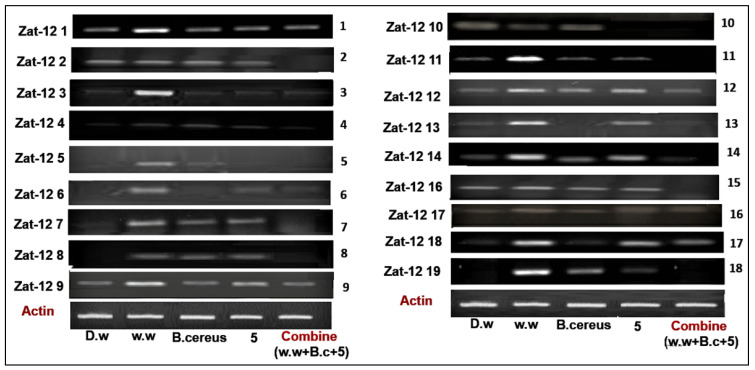
The expression profile of the zinc finger protein (Zat-12) gene family of rice plants that were grown by the synergistic treatment of bacteria-primed seeds that were grown in 5 mg/L ZnO NPs and HMs-contaminated water. The treatments are: DW (distilled water), w.w (wastewater), Bio-priming (*Bacillus cereus* + w.w), ZnO NPs treatment (5 mg/L + w.w), and combined treatments (*Bacillus cereus* + 5 mg/L ZnO NPs + w.w).

**Figure 8:**
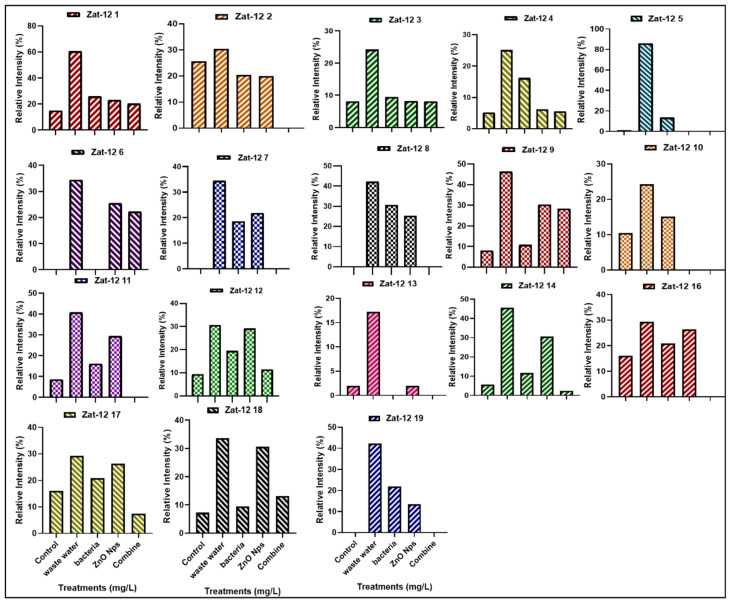
The relative intensity (%) of Zat finger protein (Zat-12) of rice plants that were grown by the synergistic treatment of bacteria-primed seeds that were grown in 5 mg/L ZnO NPs and HMs-contaminated water. The treatments are DW (distilled water), w.w (wastewater), Bio-priming (*Bacillus cereus* + w.w), ZnO NPs treatment (5 mg/L + w.w), and combined treatments (*Bacillus cereus* + 5 mg/L ZnO NPs + w.w).

The zinc finger protein (Zat-12) gene family consisting of 18 genes in rice plants work as transcription factors and play an important role in oxidative stress signaling. The present study shows the expression profile of 18 genes in various treatments, such as HMs-polluted water, *B. cereus*-primed seeds that were grown in 5 mg/L ZnO NPs, *B. cereus*-primed seeds, and individually grown at 5 mg/L ZnO NPs (Figure 7). Plants that were grown in contaminated wastewater showed 100% activity of Zat-12 genes in all gene families, with the highest expression at Zat-12-1, Zat-12-3, Zat-12-4, Zat-12-9, Zat-12-11, Zat-12-12, Zat-12-13, Zat-12-14, and Zat-12-19 with a banding intensity of 60.474, 24.154, 34.496, 46.346, 40.797, 30.651, 17.225, 45.540, 33.621, and 42.387% as compared with the distilled water grown plants with a banding intensity of 14.957, 8.100, 0.00, 7.913, 8.650, 9.320, 1.952, 45.540, 7.252%), respectively. The combined treatment of *B. cereus* seed germination that was grown in ZnO NPs resulted in a low expression of Zat-12-1, Zat-12-3, Zat-12-4, Zat-12-9, Zat-12-12, Zat-12-14, Zat-12-17, and Zat-12-18 with an intensity of 20.331, 8.100, 5.114, 28.34, 11.404, 2.448, 7.367, and 13.126% as with individual *B. cereus*-primed seeds without ZnO NPs with an intensity of 25.837, 9.479, 16.81, 46.346, 19.44, 17.588, 20.939 and 11.458%, respectively (Figure 8).

#### 3.4.3. Expression of Ascorbate Peroxidase (Apx) Gene Family

**Figure 9:**
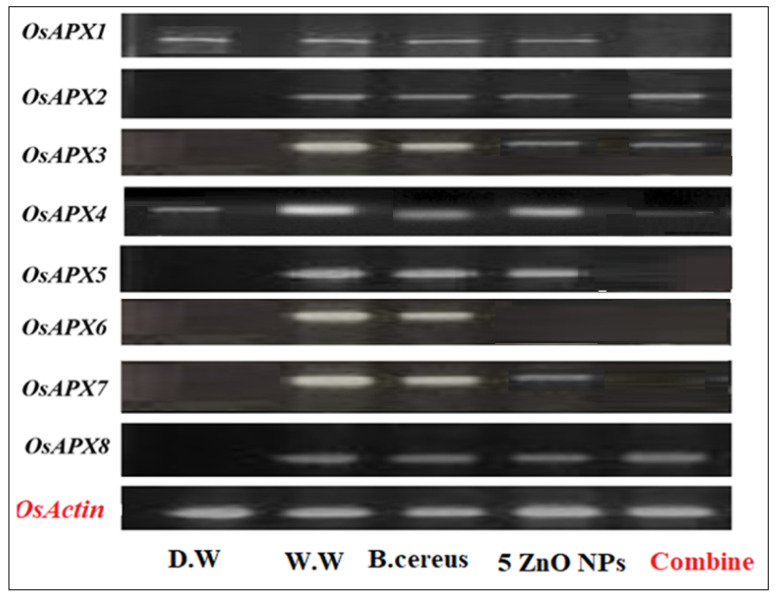
The expression profile of the Ascorbate peroxidase (APX) gene family of rice plants that were grown by the synergistic treatment of bacteria-primed seeds that were grown in 5 mg/L of ZnO NPs and HMs-contaminated water. The treatments are control (distilled water), W.W (wastewater), bio-priming (*Bacillus cereus* + w.w), ZnO NPs treatment (5 mg/L + w.w), and combined treatments (*Bacillus cereus* + 5 mg/L ZnO NPs + w.w).

**Figure 10:**
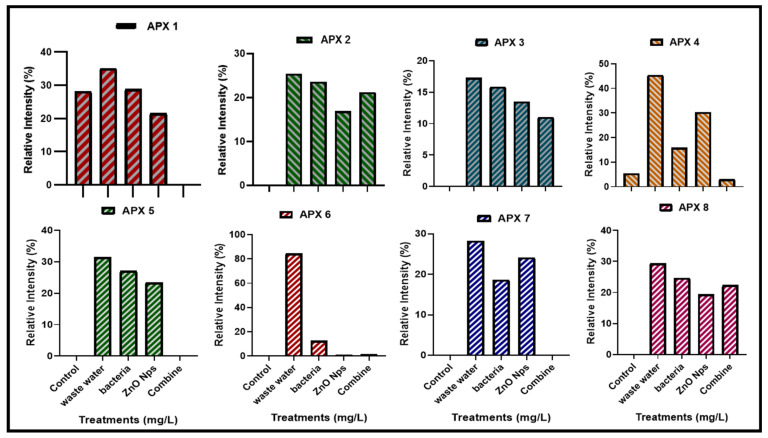
The relative intensity (%) of Ascorbate peroxidase (APX) gene family of rice plants that were grown by the synergistic treatment of bacteria-primed seeds that were grown in 5 mg/L of ZnO NPs and HMs-contaminated water. The treatments are DW (distilled water), w.w (Wastewater), bio-priming (*Bacillus cereus* + w.w), ZnO NPs treatment (5 mg/L + w.w), and combined treatments (*Bacillus cereus* + 5 mg/L ZnO NPs + w.w).

Ascorbate peroxidase (Apx) enzymes are essential in plants because they interact with Haem peroxidases and act as an electron donor to convert H_2_ O_2_ to H_2_O and O_2_. In the current research, the expression of APX genes was measured in plants that were grown in HMs-polluted water, *B. cereus*-primed seeds that were grown in 5 mg/L ZnO NPs solution, and individual *B. cereus*-primed seeds and 5 mg/L ZnO NPs (Figure 9). Rice plants that were grown in polluted water showed the highest levels of gene expression, including Apx3, Apx4, Apx5, Apx-6, and Apx7 with an intensity of 17.254, 45.420, 31.447, 84.308, and 28.236%, respectively, as compared with the control-grown (distilled water) plant (17.254%). The combined treatment of *B. cereus* seed germination of those that were grown in 5 mg/L ZnO NPs led to a lower expression of the genes Apx2, Apx-3, Apx-4, and Apx-8 with an intensity of 21.23, 11.04, 3.010, and 22.320%, respectively, as compared with plants grown by individual *B. cereus*-primed seeds without ZnO NPs with an intensity of 23.601, 15.841, 15.866 and 24.662%, respectively (Figure 10).

## 4. Discussion

The rapid increase in industrialization and anthropogenic activities add a huge amount of HMs to water that is subsequently used for irrigation and drinking purposes. The solubilized form of HMs cannot be easily removed from water [33,34]. Plant growth is strongly influenced by the proteomic and molecular changes that occur due to HM stress [35,36]. Therefore, there is a growing demand to use adsorbents of higher efficiency to remove HMs. Bacteria and nanoparticles have gained vast attention due to their morphological, textural, and structural properties [37]. Bacteria secrete biofilm and exopolysaccharides having negative charges that bind with the HMs and NPs [38]. Bacteria secretes amino cyclo-propane (1-carboxylic acid (ACC) deaminase and bioactive metabolites such as surfactant and lipo-peptides that protect the seeds from abiotic stress and increase the germination [11]. Previously, nanoparticles, particularly, ZnO NPs have been utilized for their role in anti-microbial activities [39,40]. However, to our knowledge, a very limited literature is available about the potential role of ZnO NPs in the enhancement of HMs-remediation by bacteria in rice plants. ZnO NPs have been reported to improve growth by boosting the nutrient reserves and improving molecular activities in HMs stress conditions [41]. Additionally, Zn^+2^ ions from ZnO NPs are necessary for bacterial enzyme (dehydrogenase, thiol peroxidase, and glutathione reductase) functions, which improve their growth by boosting the nutrient reserves, proteomic, and molecular activities [42]. Furthermore, at the same time, these zinc ions enhance the cell stability by directly enhancing the nutrition uptake, water molecules conduction from the roots to the upper parts of the plant and increase the resistance level of the plant against HMs [43]. Therefore, in the current study we aimed to find the potential role of ZnO NPs in the remediation of HMs by bacteria-primed seeds that were grown in HMs-contaminated water and initially looked for the changes in protein and gene level.

It was observed that the nitrogen-protein content exhibited a decrease in plant tissues (leaf, shoot, and root) (Table 3). Basically, plants utilize nitrogen by reducing the inorganic form of nitrogen such as nitrate and ammonia into organic nitrogen (urea and amino acid). As such, toxic metal ions affect the plant by causing a reduction of nitrogen metabolism and protein synthesis [44]. These results showed similarities with the findings of Zhou [12], who showed that HMs lower the nitrogen-protein metabolism in plants. HMs increased the photorespiration in plants by causing oxidation and modification in the tertiary structure and function of proteins [45]. It was investigated by Kerchev [10] that cadmium toxicity causes oxidative stress in the *Brassica juncea* plant, by binding with the active site of cysteine residue of the protein and changes beta carbonic anhydrides. On the other hand, seeds that were primed with bacteria and grown in ZnO NPs enhanced the concentration of total the protein-nitrogen content and remediated the HM stress (Table 3). Plants also release bioactive signal molecules such as nitric oxide and reduce the destructive action of HMs [46]. Bacterial priming increased the growth of a plant by enhancing nutrients, phosphate solubilization, photosynthesis, and modulation of ACC deaminase activity [7]. It was investigated that Zn ions from ZnO NPs act as essential nutrients for many biochemical functions in plants such as producing chlorophyll, carbohydrates, and nitrogen metabolism [36]. Similar results were reported by Zhou [12] that TiO_2_ and SiO_2_ improved soybean (*Glycine max*) germination by increasing the nitrate reductase activity and enhancing the absorption capacity in the plants. The maximum amount of nitrogen and protein content was due to the immobile nature of metals in water which results in lower the oxidation state of nitrogen and protein [43].

The expression of the Myb genes families was enhanced in plant growth in HMs-contaminated water (Figure 5 and Figure 6). These results showed similarity with the findings of Carrouel [38], that expression of Myb genes activate the stress signaling in plants under drought conditions. It was also investigated [10], that there was an overexpression of the RsMYB1 gene in the transgenic petunia plant under HMs stress. The results revealed that seeds that were primed with *B. cereus* and *L. macroides* and grown in ZnO NPs showed a lower expression of genes (Figure 5 and Figure 6). It was reported by Sabir [46] that silver NPs size and shape affects the growth and expression of genes in *Arabidopsis thaliana* plants.

The Zat-12 gene increased in plants under abiotic stress, wound-induced systemic signals, and oxidative- and heat-related stresses [46]. The results showed that zinc finger protein (Zat) gene expression up-regulated in plants that were grown in the HMs-polluted water (Figure 7 and Figure 8). Similar results were observed by Fan [20], that zinc finger protein genes were also activated in the plants under reactive oxygen species and lipid peroxidation stress which causes decreased cell growth and biogenesis, including microtubule-based processes and nucleosome assembly. There was variation of the zinc finger protein gene expression under hypoxia stress in rice plants [15]. The results revealed that gene expression was lowered in combined treatments of bacteria and ZnO NPs (Figure 7 and Figure 8). A recent report has been studied by Hossain [10] that metals that are resistant endophytic bacteria reduce the toxicity of cadmium and nickel by the decreased the expression of stress-related genes and further increased rice (*Oryza sativa* L.) growth by regulating antioxidant machinery and endogenous hormone synthesis and causes detoxification of metals. ZnO NPs reduce the flow of metals by decreasing the accumulation of metals and lower the expression of stress-induced genes [20]. It was studied that ZnO NPs and titanium oxide nanoparticles’ effect on *Arabidopsis thaliana* (L.) plants showed down-regulated expression of stress-related genes under biotic and abiotic stress [45].

HMs produce oxidative stress in plants by generating different scavenging enzymes in which ascorbate peroxidases (APX) are enzymes that scavenge oxidative stress. The results showed that the expression of APX genes was maximum in plants that were under HMs stress (Figure 9 and Figure 10). It was reported by Fan [20], that APX gene expression in plants was increased in HMs stress conditions. It was observed that the synergistic treatment of bacteria and nanoparticles removed the HMs from water and showed less expression of the APX gene (Figure 9 and Figure 10). It was investigated by Gonzalez [47], that priming with plant growth-promoting bacteria downregulated stress-related genes (APX1, SAMS1) under drought stress in wheat plants. These results showed similarity with the finding of Gonzalez [48,49] that priming of seeds with bacteria decreased the expression of stress-related genes in rice (*Oryza sativa* L.) under salt stress. The NPs size and shape affect the expression of APX genes such as ZnO. NPs treatment in plants showed both upregulation and downregulation of different genes [50]. It was reported that silver NPs size and shape affect the growth and expression of APX genes in *Arabidopsis thaliana* [51]. Similar results were observed by the findings of Gupta [52,53] which showed that APX gene expression was different in rice plants that were grown by foliar spray of silver nanoparticles treatment. It is highly recommended to develop and subsequently, commercialize bio-nano-remediation processes which are ultimately required for sustainable environmental protection. Further research is needed to elaborate the interaction of ZnO NPs with bacteria and subsequently increase their efficiency to rehabilitate the existing heavy metals-contaminated water.

## 5. Conclusions

This study is a new insight as it explores how the supplementation of lower concentrations of ZnO NPs interaction with bacteria may regulate the remediation mechanism at proteomic and molecular levels. Rice, a non-tropical plant, has been explored for its response to HMs-contaminated water, not only in terms of nitrogen uptake but also protein in plant tissues (root, shoot, and leaf) that are grown by primed bacterial seeds supplementing the hydroponic culture tray with lower concentrations of ZnO NPs. This study demonstrated the novel findings depicting the positive effect of bacteria-ZnO NPs combined treatment on the protein profile of plants in SDS-PAGE analysis. The study also paved the way for exploring the role of the ZnO NPs-bacteria interaction altering the stress-induced gene expression (MYB, Zat-12, and APX) and the intensity of bands in HMs-contaminated water. Exploring the role of the synergistic treatment of bacteria-ZnO NPs regulating protein changes may provide the impetus to regulate stress-induced gene expression.

## Figures and Tables

**Table 1:** Chemical composition of gel for running and stacking.

Running Gel Composition		Stacking Gel Composition	
Components	Quantity	Components	Quantity
1.5 M Tris-HCl (pH 8.8)	2.6 mL	0.5 M Tris-HCl (pH 6.8)	2.50 mL
Distilled water (DW)	3.2 mL	Distilled water (DW)	3.975 mL
30% of Acrylamide	4 mL	30% of Acrylamide	600 µL
20% of SDS	100 µL	10% of APS	50 µL
10% of APS	100 µL	20% of SDS	100 µL
TEMED	7–10 µL	TEMED	5–7 µL

**Table 2:** The physico-chemical parameters of the Hayatabad industrial estate (HIE) were analyzed to evaluate the pollution load and HMs content in water and compare it with permissible limit of National Environment Quality Standard (NEQS) for industrial effluents.

Physico-Chemical Parameters	HIE Wastewater	Standard (NEQS)
Temperature	24.5 °C	40.0 °C
pH	7.23 pH	6–10 pH
EC	682 (µS/m)	500 (µS/m)
Odure	Bad	Bad
TSS	400 mg/L	150 mg/L
TDS	4485 mg/L	3500 mg/L
BOD	250 mg/L	80 mg/L
COD	400 mg/L	150 mg/L
Lead	2.84 mg/L	0.5 mg/L
Cadmium	2.26 mg/L	1.0 mg/L
Chromium	2.40 mg/L	1.0 mg/L
Iron	1.19 mg/L	0.2 mg/L
Manganese	1.36 mg/L	1.5 mg/L
Nickle	1.83 mg/L	1.0 mg/L

NEQS: National environmental quality standards, HIE: Hayatabad industrial estate, EC: Electrical Conductivity, TSS: Total suspended solids, TDS: Total dissolved solids, BOD: Biochemical oxygen demand, COD: Chemical oxygen demand.

**Table 3:** The total nitrogen-protein contents in the leaf-shoot-root of rice plants that were grown under synergistic treatment of seeds that were primed with bacteria strains (*Bacillus cereus* and *Lysinibacillus macroides*) and grown in ZnO NPs (5 and 10 mg/L) alone or in combination with HMs-contaminated water. Control (distilled water), B.C + 5 mg/L (*Bacillus cereus* + 5 mg/L ZnO NPs) and L.M + 5 mg/L (*Lysinibacillus macroides* + 5 mg/L ZnO NPs), B.C + 10 mg/L (*Bacillus cereus* + 10 mg/L ZnO NPs) and L.M + 10 mg/L (*Lysinibacillus macroides* + 10 mg/L ZnO NPs). Values are the mean (±) standard deviation of three replicates (*n* = 3) followed by different alphabetic letters showing statistical significance at 5% probability level (ANOVA and Duncan’s multiple range test).

	Total Nitrogen (µg/g)						Total Protein (µg/g)					
Treatments	Leaf		Shoot		Root		Leaf		Shoot		Root	
	Mean	SD	Mean	SD	Mean	SD	Mean	SD	Mean	SD	Mean	SD
D.W	5.62	0.211 ^a^	4.23	0.029 ^ab^	3.11	0.023 ^a^	45.43	0.026 ^de^	40.43	0.01 ^a^	32.22	0.028 ^ab^
Wastewater	2.21	0.029 ^a^	1.62	0.064 ^cde^	1.12	0.052 ^a^	15.32	0.031 ^ad^	9.23	0.054 ^a^	6.21	0.01 ^bc^
*Bacillus cereus*	12.8	0.034 ^ab^	9.31	0.021 ^ab^	6.12	0.028 ^ab^	60.23	0.015 ^cd^	53.23	0.023 ^ab^	47.31	0.078 ^bc^
(*B. cereus* + w.w)	14.32	0.039 ^abc^	10.52	0.083 ^cdf^	8.23	0.011 ^ab^	23.21	0.023 ^ab^	16.43	0.063 ^ab^	10.23	0.016 ^a^
*Lysinibacillus macroides*	10.2	0.020 ^a^	7.22	0.037 ^ac^	5.43	0.078 ^bc^	55.41	0.012 ^ab^	47.41	0.021 ^abc^	35.41	0.078 ^b^
(L. *macroides* + w.w)	12.13	0.018 ^ab^	8.32	0.094 ^abc^	6.43	0.024 ^ab^	21.12	0.01 ^ab^	14.12	0.034 ^b^	9.23	0.025 ^c^
5 mg/L ZnO Nps	15.21	0.018 ^a^	10.31	0.022 ^a^	8.26	0.037 ^def^	62.21	0.013 ^ab^	47.32	0.012 ^ab^	38.23	0.030 ^a^
(5 mg/L + w.w)	12.23	0.017 ^bc^	7.21	0.032 ^cdf^	5.46	0.015 ^ac^	22.34	0.021 ^a^	16.54	0.051 ^bc^	10.78	0.052 ^c^
10 mg/L ZnO Nps	20.31	0.039 ^d^	15.21	0.025 ^bc^	11.61	0.030 ^ab^	67.12	0.022 ^ab^	50.21	0.043 ^ab^	42.12	0.017 ^c^
(10 mg/L + w.w)	17.21	0.039 ^bcd^	12.42	0.074 ^ab^	9.62	0.027 ^ab^	34.21	0.021 ^ab^	28.23	0.012 ^ab^	23.21	0.017 ^b^
(*B. cereus* +5)	20.23	0.037 ^b^	18.31	0.01 ^ab^	14.81	0.091 ^bc^	70.32	0.024 ^ab^	63.21	0.011 ^abc^	56.21	0.052 ^ab^
(*B. cereus* +5 + w.w)	25.32	0.013 ^bcd^	20.43	0.073 ^abd^	17.62	0.032 ^ab^	32.21	0.034 ^b^	26.54	0.032 ^c^	16.43	0.032 ^a^
(*L. macroides* +5)	19.9	0.023 ^ab^	15.41	0.016 ^ae^	12.13	0.056 ^be^	66.41	0.021 ^ab^	52.31	0.032 ^a^	47.32	0.015 ^ab^
(*L. macroides* +5 + w.w)	22.65	0.022 ^abc^	17.22	0.047 ^abe^	15.51	0.016 ^ab^	30.22	0.043 ^ab^	22.21	0.021 ^bd^	14.21	0.016 ^c^
(*B. cereus* +w.w)	27.52	0.024 ^bc^	22.12	0.062 ^bc^	19.72	0.033 ^ab^	77.22	0.03 ^ab^	45.32	0.024 ^b^	49.32	0.013 ^cd^
(*B. cereus* + 10 + w.w)	21.42	0.031 ^abc^	18.32	0.026 ^ab^	17.73	0.019 ^ac^	42.54	0.04 ^ab^	31.21	0.024 ^ab^	29.43	0.013 ^bc^
(*L. macroides* +w.w)	26.24	0.025 ^ab^	20.32	0.022 ^ab^	15.52	0.094 ^ab^	70.31	0.06 ^ab^	39.12	0.031 ^bc^	53.23	0.022 ^b^
(*L. macroides* +10 + w.w)	22.31	0.034 ^abd^	13.32	0.072 ^ab^	15.62	0.017 ^ab^	40.21	0.03 ^ab^	30.42	0.032 ^c^	26.43	0.022 ^a^

## Data Availability

Not applicable.
